# An episignature informed systematic analysis to ascertain the clinical significance and consequences of *CHD8* missense variants

**DOI:** 10.1038/s41431-026-02209-4

**Published:** 2026-08-10

**Authors:** Molly Godfrey, Michael A. Levy, Christopher Campbell, Leigh Demain, Sarah Jenkinson, Sarah Hilton, Berta Almoguera, Meena Balasubramanian, Emilia K. Bijlsma, Fiona Blanco-Kelly, Emma M. M. Burkitt Wright, Gerarda Cappuccio, Kate E. Chandler, Koen Devriendt, Aranzazu Diaz de Bustamante, Maria K. Haanpää, Carolin Hörnig, Elizabeth A. Jones, Sinem Kocagil, Hannele Koillinen, Dhanya Lakshmi Narayanan, Emanuela Leonardi, Rajesh Madhu, Purvi Majethia, Alessandra Murgia, Elisabeth Rosser, Markus Schuelke, Anju Shukla, Emma Soengas-Gonda, Sarah Stewart, Yves Sznajer, Saoud Tahsin Swafiri, Maria Margarida Venancio, Renzo Guerrini, Martino Montomoli, Annalisa Vetro, Thomas Wright, David Gokhale, Bekim Sadikovic, Sofia Douzgou Houge, Siddharth Banka

**Affiliations:** 1 Manchester Centre for Genomic Medicine, St Mary’s Hospital, Manchester University NHS foundation Trust, Health Innovation Manchester, Manchester, UK; 2 North West Genomic Laboratory Hub (Liverpool site), Liverpool Women’s Hospital, Crown Street, Liverpool, UK; 3 Verspeeten Clinical Genome Centre, London Health Sciences Centre, and Department of Pathology and Laboratory Medicine, Western University, London, ON Canada; 4 Department of Pathology and Laboratory Medicine, Western University, London, ON Canada; 5 Centro de Investigacion Biomedica en Red de Enfermedades Raras (CIBERER), Instituto de Salud Carlos III (ISCIII), Madrid, Spain; 6 Department of Genetics and Genomics, Fundación Jiménez Díaz University Hospital. Health Research Institute-Fundación Jiménez Díaz University Hospital, Universidad Autónoma de Madrid (IIS-FJD, UAM), Madrid, Spain; 7 Sheffield Clinical Genomics Service, Sheffield Children’s NHS Foundation Trust, Sheffield, UK; 8 Division of Clinical Medicine, School of Medicine and Population Health, University of Sheffield, Sheffield, UK; 9 Department of Clinical Genetics, Leiden University Medical Centre, Leiden, The Netherlands; 10 Division of Evolution, Infection and Genomics, School of Biological Sciences, Faculty of Biology, Medicine and Health, University of Manchester, Manchester, United Kingdom; 11 Department of Translational Medicine, Section of Pediatrics, Federico II University, Naples, Italy; 12 Telethon Institute of Genetics and Medicine, Naples, Italy; 13 Centre for Human Genetics, University Hospital, University of Leuven, Leuven, Belgium; 14 Unidad de Genetica, Hospital Universitario de Mostoles, Mostoles, Spain; 15 Department of Clinical Genetics, Turku University Hospital, Turku, Finland; 16 Department of Genomics, Turku University Hospital, Turku, Finland; 17 Department of Neuropediatrics, Jena University Hospital, Jena, Germany; 18 Department of Medical Genetics, Eskisehir Osmangazi University Faculty of Medicine, Eskisehir, Turkey; 19 Department of Medical Genetics, Kasturba Medical College, Manipal, Manipal Academy of Higher Education, Manipal, India; 20 Department of Biomedical Sciences, University of Padova, Padova, Italy; 21 Department of Woman and Child Health, University of Padova, Padova, Italy; 22 Department of Neurosciences, Alder Hey Children’s NHS Foundation Trust, Liverpool, UK; 23 Department of Clinical Genetics, Great Ormond Street Hospital for Children, London, UK; 24 NeuroCure Cluster of Excellence, Charité-Universitätsmedizin Berlin, Corporate Member of Freie Universität Berlin, Humboldt-Universität zu Berlin and Berlin Institute of Health, Berlin, Germany; 25 Department of Neuropediatrics, Charité-Universitätsmedizin Berlin, Corporate Member of Freie Universität Berlin, Humboldt-Universität zu Berlin and Berlin Institute of Health, Berlin, Germany; 26 Unidad de Dismorfología y Genetica (UDisGen), Hospital Universitario 12 de Octubre, Madrid, Spain; 27 Center for Human Genetics Cliniques Universitaires Saint-Luc UCLouvain, Brussels, Belgium; 28 Department of Genetics, Hospital Pediátrico, Centro Hospitalar e Universitário de Coimbra, Coimbra, Portugal; 29 Medical Genetics Institute - UC Genomics, Faculty of Medicine, University of Coimbra, Coimbra, Portugal; 30 Pediatric Neurology, Neurogenetics and Neurobiology Unit and Laboratories, Meyer Children’s Hospital, University of Florence, Florence, Italy; 31 Department of Neuroscience, Psychology, Drug Research and Child Health, University of Florence, Florence, Italy; 32 Department of Medical Genetics, Haukeland University Hospital, Bergen, Norway; 33 Frambu Resource Centre for Rare Disorders, Siggerud, Norway

**Keywords:** Clinical genetics, Genomics, Epigenomics

## Abstract

Pathogenic *CHD8* variants cause autosomal dominant ‘Intellectual developmental disorder with autism and macrocephaly’ (IDDAM) and are amongst the most common monogenic causes of autism. The clinical significance of *CHD8* missense variants (MVs) frequently remains uncertain. Systematically applying ACGS/ACMG guidelines to 36 *CHD8* MVs in 39 affected patients, only two variants were classified likely pathogenic (LP), with the remaining 34 classified variants of uncertain significance (VUS). We subclassified the variants according to posterior probability of pathogenicity (PPP), with 14 being at least tepid VUS (PPP ≥ 50%). Comprehensive phenotypic analysis revealed no discernible clinical differences between individuals carrying at least tepid VUS and others, offering no additional insight for variant classification. EpiSign™ testing revealed a *CHD8*-IDDAM episignature, as previously detected in patients with truncating/null variants, in 11 cases, allowing reclassification of 8 VUS (all previously classified at least tepid) as LP. Molecular modelling indicated that disease-causing (LP/P) *CHD8* MVs are concentrated in structured and/or functional protein domains. Compared to truncating/null variants, disease-causing MVs were less often associated with attention issues and macrocephaly, but clinical features were otherwise similar. Additionally, three disease-causing MVs were inherited from unaffected/mildly affected parents. Overall, we show that determining the clinical significance of *CHD8* MVs is challenging, even with detailed clinical information, but that incorporating episignature analysis increases diagnostic yield. Further, our results indicate that *CHD8* MVs are likely to act via a loss-of-or reduced function mechanism. These findings reveal the importance and complexity of interpreting *CHD8* MVs and will improve the diagnosis and understanding of *CHD8*-related disorders.

## Introduction

Members of the chromohelicase domain (*CHD*) family of nine highly conserved epigenetic regulators in humans affect chromatin compaction and control multiple critical biological processes, such as transcription, cellular proliferation, and DNA damage repair. All CHD proteins share two, modified, histone-binding chromohelicase domains and a bipartite ATPase motor composed of an SNF2-like and a helicase-C (HELIC-C) domain, that together facilitate nucleosome mobilization. Germline variants in *CHD* genes cause a range of monogenic disorders [1–6]. *CHD8* belongs to CHD Subfamily III and heterozygous, germline, loss-of-function (LoF) variants in this gene cause ‘Intellectual developmental disorder with autism and macrocephaly’ (IDDAM, previously known as ‘AUTS18’, OMIM# 615032) [7–9]. *CHD8-*IDDAM is characterised by intellectual disability (ID), autism spectrum disorder (ASD), attention deficit hyperactivity disorder (ADHD), sleep problems, seizures and hypotonia [8–14]. *CHD8* variants are found in about 0.5% of individuals with ASD, suggesting that *CHD8-*IDDAM may be one of the most common monogenic forms of autism [7, 8].

Other frequent features of *CHD8-*IDDAM include overgrowth and gastrointestinal difficulties. Affected individuals also tend to share a distinctive gestalt. However, some affected individuals may not display the typical features of the condition and the phenotype can be highly variable even within the same family [11]. The variable and non-specific clinical features of *CHD8-*related IDDAM hamper both the clinical diagnosis and reverse phenotyping of *CHD8*-related IDDAM [15]. Interestingly, *CHD8* LoF patient cohorts are enriched for affected males, suggesting possible sex-dependent penetrance for the disorder although this remains to be proven conclusively [12, 13, 16]. Existing evidence has been overwhelmingly focused on patients with truncating variants [12, 13, 16]. It is recognised that the severity of disorders caused by truncating/null variants and missense variants (MVs) in genes can differ. Therefore, even if a *CHD8* MV is classified as pathogenic, providing prognostic information can be difficult because the severity and penetrance of the disorder caused by *CHD8* MVs has not been systematically investigated.

In recent years, a growing number of episignatures have been implemented in clinical diagnostic laboratories as diagnostic biomarkers of congenital genetic disorders and for the reclassification of variants of uncertain significance (VUSs) [17–21]. Episignatures have also been shown to correlate with phenotype [22]. An episignature for null/truncating (presumed to undergo nonsense mediated decay and therefore act as loss of function - LoF) *CHD8* variants has been established and integrated in the EpiSign™ diagnostic pipeline (CHD8-IDDAM-specific) [20, 22].

Here, we show that clinical interpretation of *CHD8* MVs is challenging, and present results of an episignature-based genotype-phenotype study, demonstrating that episignature profiling improves the accuracy of diagnosis in *CHD8*-IDDAM. We also provide insights into the pathogenicity of *CHD8* MVs by showing similar episignature profiles as known LoF variants and that disease-causing *CHD8* MVs are enriched in highly structured and/or functionally important protein domains, supporting a reduced function or loss-of-function mechanism. Using comparative phenotypic analyses, we show that in comparison with known LoF variants, *CHD8* (likely) pathogenic MVs tend to result in similar, but potentially milder clinical presentation. Finally, we show that disease-causing *CHD8* variants can result in incomplete penetrance and can present pleiotropically.

## Materials and methods

### Variant compilation, and patient ascertainment

*CHD8* LoF (nonsense and frameshift - truncating) variants and MVs were downloaded from ClinVar, Human Gene Mutation Database (HGMD) and DECIPHER, and grouped by clinical significance as described in the databases [23–25]. A call for individuals with rare *CHD8* MVs was disseminated through the ERN-ITHACA network mailing list [26] and website, as well as in national and international genetic syndromology meetings. Participating clinicians were provided with a blank version of the excel spreadsheet submitted as Supplementary Tables S2, S3, to ensure standardised collection of phenotype information. Informed consents for the use and publication of medical data and biological material were obtained from all affected individuals or their legal representative by their clinician. Consents for publication of photographs were obtained.

### Variant classification

All the variants were classified according to the Association for Clinical Genomic Science/American College of Medical Genetics (ACGS/ACMG) guidelines for SNV classification [27, 28]. The variants of uncertain significance (VUS) were further subdivided according to pathogenicity status according to the ACGS guidelines and their statistical posterior probability of pathogenicity [28–30]. REVEL scores were used for in silico predictions of pathogenicity with PP3 applied to variants with a score of ≥0.7. All variants with REVEL scores of <0.7 were also analysed for their predicted impact on splicing using SpliceAI [31]. BP4 was applied to variants with a REVEL score ≤0.4 and no predicted impact on splicing. *CHD8* has a gnomAD (v.4.1.1) missense constraint score of 8.14 (>3.09 threshold), which would allow application of PP2 at the gene level according to ACMG/ACGS guidelines. Regional and residue-level missense constraint were also considered by combining information from the DECIPHER regional missense constraint score and the missense tolerance ratio (MTR-viewer) [32, 33]. PP2 was applied to variants in the DECIPHER region of high missense constraint (aa 630–1727) if they had an MTR-score consistent with being under purifying selection (<1), and to variants outside the DECIPHER region of high missense constraint if they had an MTR-score <0.75 (below the median score) [32, 33]. PM1 was applied to variants in the HELIC-C domain, within which missense variants have been demonstrated to be enriched in patients with ASD [34]. Insufficient evidence was available to allow for application of PM1 to variants in any of the other known functional protein domains. PS2/PM6 was applied to de novo variants (grouped under category PS2 for simplicity whether or not parental identity has been confirmed, but following the Clingen SVI recommendations and points system), downgraded to a supporting level of evidence due to *CHD8*-associated phenotypes being both non-specific and heterogeneous. PM2 was applied to variants with a MAF < 3 × 10^−6^ in gnomAD v4.1.0 to allow for the potential for incomplete penetrance.

The presence of a high confidence DNA methylation signature in a patient with a variant in the gene associated with the identified signature is considered to a ‘strong’ piece of evidence as per ACGS 2024 guidelines and international reporting recommendations [28, 35]. As per recommendations DNAm evidence was integrated into ‘phenotype-specificity’ criteria PP4 or PS2/PM6 (the latter if de novo status known) post DNA methylation analysis using EpiSign™. The evidence strength was downgraded to a moderate level for variants with a moderate confidence DNA methylation signature. Fisher’s exact test (two-tailed) was used for statistical analyses of correlation between posterior probability of pathogenicity and presence of a DNAm signature.

### DNA methylation signature analysis

Test samples were compared to the previously reported AUTS18/CHD8 (CHD8) episignature [22] using R v4.2.2. IDAT files were processed and normalized using minfi v1.44.0 to generate the methylation beta values used for subsequent analysis. Methylation Variant Pathogenicity (MVP) scores were generated using the R package e1071 v1.7-12 by converting SVM decision values to probability scores using Platt’s scaling method. Multidimensional scaling (MDS) used the CHD8 episignature probes as features and was performed by first scaling of the pair-wise Euclidean distances between samples. Hierarchical clustering and heatmaps were generated with the R package gplots 3.1.3 using the ward.D method for clustering. High and moderate confidence was assigned to episignatures as defined as described previously [35].

### Phenotype analysis

Comparative genotype-phenotype analysis was performed, using phenotypic data from this cohort and the previously published cohorts of individuals with *CHD8* variants [12, 16]. Fisher’s exact test (two-tailed) was used for statistical analyses. *p*-values were calculated with and without multiple correction, the latter using the Benjamini–Hochberg false discovery rate (FDR) method, applied separately for each comparison. Effect sizes were estimated using odds ratios (ORs) with 95% confidence intervals (CIs), calculated from 2 × 2 contingency tables using the log method.

### Mutation modelling

The 3D structure prediction for the protein (in PDB format) was obtained from the AlphaFold protein structure database [36] and imported into PyMoL v.2.5.3 where it was visualised [37]. Protein chains were coloured according to functional domains as predicted in ref. [34] and location of the MVs plotted onto the predicted 3D structure

## Results

### Clinical interpretation of CHD8 MVs is challenging

We ascertained 558 *CHD8* variants (106 loss of function (LoF) and 452 missense variants (MVs)) from ClinVar [25], 205 (93 LoF and 112 MVs) from HGMD [23] and 25 (12 LoF and 13 MVs) from DECIPHER [24] (Supplementary Fig. S1). Overall, 62–100% of LoF *CHD8* variants were classed as Pathogenic, Likely Pathogenic or Deleterious Mutation (P/LP/DM) in these databases. In contrast, 77–82% *CHD8* MVs across the three databases were annotated as of uncertain significance.

A recent study reported phenotypic analysis of 106 individuals with *CHD8* variants, including 27 with MVs and 2 patients with in-frame single amino acid deletions [16]. In this study, although all individuals were stated to have ‘(likely) pathogenic’ *CHD8* variants, the variants were not systematically classified. We performed ACGS/ACMG guidelines-based classification for all missense variants reported in the paper and show three (12%) variants could be classified as likely pathogenic and 23 (88%) as VUS [27, 28]. Four of the VUS (15% of the total variants) had in silico evidence against pathogenicity (Supplementary Fig. S1, Supplementary Table S1).

**Fig. 1:**
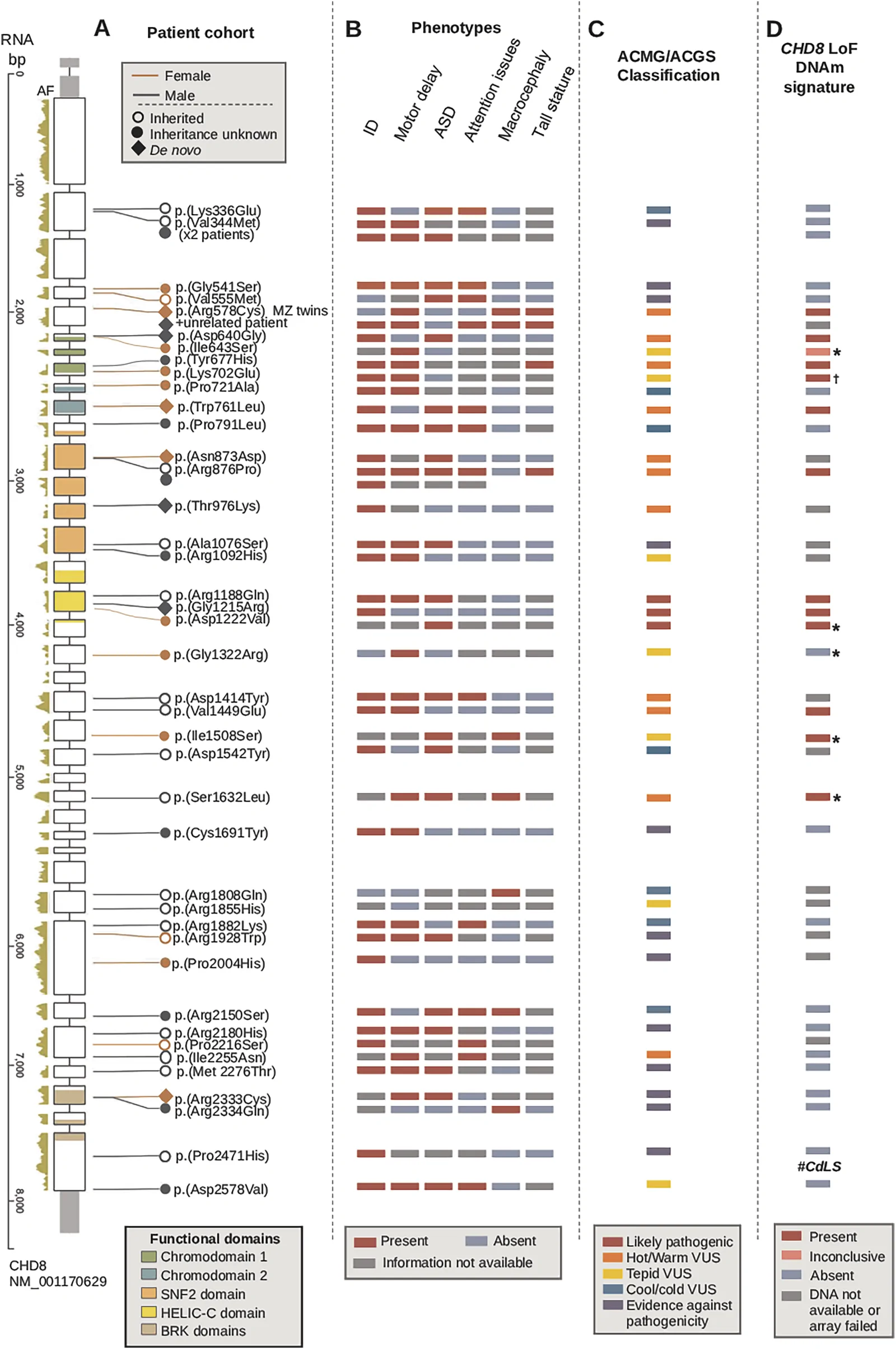
Systematic classification of *CHD8* missense variants and the role of episignatures. **A** Distribution of 41 *CHD8* missense variants along the CHD8 protein, including 36 variants from 39 patients gathered for this study and 5 previously published patients who previously had DNAm analysis performed according to the EpiSign pipeline [16] (*). Nomenclature provided refers to the protein change with full HGVS nomenclature for each variant provided in Supplementary Tables S1 and S4. †Moderate confidence signature. Exon structure of the mRNA is indicated along with known functional protein domains. gnomAD missense variant allele frequency is indicated for each exon (AF), as displayed in DECIPHER. **B** Phenotypic analysis of patients. **C** ACMG/ACGS classification status of variants. **D** DNA methylation signature positive and negative samples.

Next, through our collaborative network, we assembled a cohort of 39 clinically affected probands (27 male and 12 female, 34 previously unreported) from 38 families, with 36 distinct rare *CHD8* MVs. In 9 individuals the variants had arisen de novo, in 19 individuals the variants were inherited from an apparently unaffected or mildly affected parent and in 11 individuals the inheritance information was unavailable (Fig. 1A, B, Supplementary Tables S2, S3). These MVs were represented throughout the gene (Fig. 1A). ACGS/ACMG guidelines-based classification of variants, including a Bayesian framework based sub-categorisation of VUS, showed that only two variants (6%) could be classified as likely pathogenic, three (9%) as hot VUS (posterior probability of pathogenicity, PPP ≥ 81.2%), seven (19%) as warm VUS (PPP ≥ 67.5%), four (11%) as tepid VUS (PPP ≥ 50%), three (8%) as cool VUS, and three (8%) as cold VUS (Fig. 1C, Supplementary Table S1) [27–30]. Fourteen variants (39%) had evidence against pathogenicity, but this evidence was insufficient for a likely benign or benign classification. We grouped the 16 likely pathogenic, hot, warm or tepid variants (PPP ≥ 50%) together, and the remaining 20 variants (PPP < 50%) separately. To investigate if phenotype information could help clarify the significance of the variants, we collected clinical information via a standardised proforma (Supplementary Table S2). This showed that out of 39 probands, 31 (80%) had some degree of intellectual disability, 31 (80%) were reported to have language delay, 24 (62%) had a history of motor delay, 20 (62%) had a diagnosis of autism spectrum disorder, 11 (28%) had coordination issues, 6 (15%) had macrocephaly, 6 (15%) had tall stature, and several had a variety of neurological and behavioural attributes (Fig. 1B, Supplementary Table S2). Importantly, there were no obvious differences in clinical presentation between the patients with at least tepid VUS and others (Supplementary Fig. S2).

Collectively, these data show that ascertaining the clinical significance of *CHD8* MVs is challenging, even with detailed clinical information.

### DNA methylation episignature analysis improves classification of *CHD8* MVs and reveals likely molecular mechanism

We performed DNAm analysis using the EpiSign™ assay on 27/39 individuals (including a pair of monozygotic twins) from our cohort and where peripheral blood DNA samples were available. Episignature analysis of the 27 MV samples revealed a high confidence *CHD8*-IDDAM specific episignature in ten (37%), a moderate confidence episignature in one (4%) and negative in 16 (59%) samples (Fig. 1D, Supplementary Fig. S3). The episignatures of patients with missense variants were not found to cluster separately or show any significant differences, compared to those of patients with truncating/loss of function variants previously analysed by the EpiSign™ pipeline, indicating a likely shared molecular mechanism (Supplementary Fig. S3).

A review of published literature revealed that episignature analysis had been previously described in five individuals with *CHD8* MVs [16] (highlighted by * in Fig. 1D). Of these, high confidence episignatures were detected in three patients, and an inconclusive and a negative signature in one patient each [16].

**Fig. 2:**
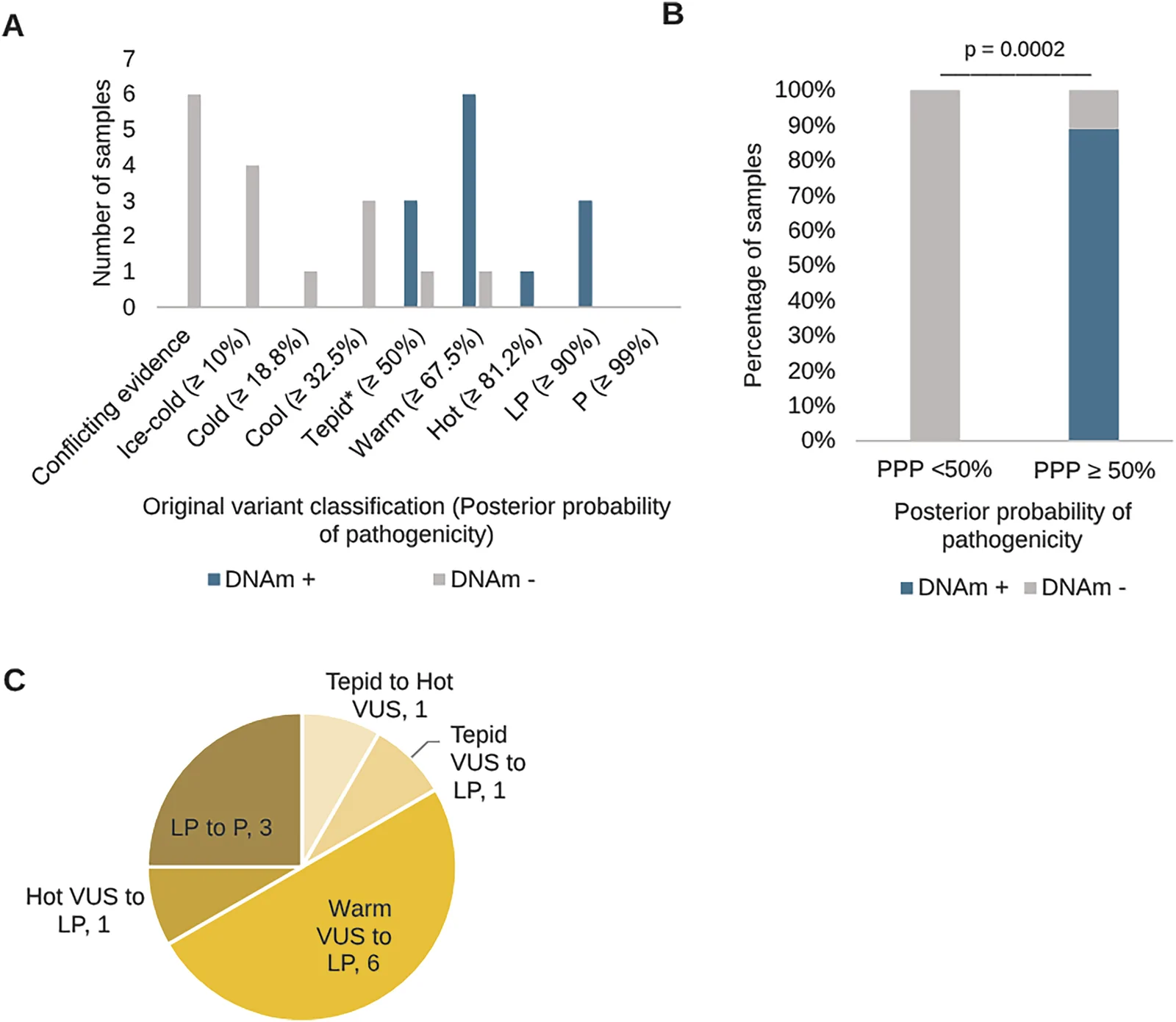
Correlation between ACGS/ACMG classification and episignature results. **A** Number of samples in each VUS ‘category’ (subdivided by temperature scale and prior probability of pathogenicity) which tested positive and negative for the *CHD8*-IDDAM episignature. *One ‘tepid’ VUS was inconclusive so was not included in either the positive or negative category, and one positive was ‘moderate’ confidence only but was included in the positive category. **B** Correlation between posterior probability of pathogenicity and episignature presence, subdividing variants into categories of <50% PPP and ≥50% PPP. *p* value was calculated using Fisher’s exact *t*-test. **C** Change in variant classification post episignature analysis for DNAm + samples. VUS Variant of uncertain significance, LP Likely pathogenic, P Pathogenic.

Comparison of the episignature results with our initial ACGS classification predictions (including the variants from ref. [16]) showed that all the MVs with a positive *CHD8* episignature were initially classified as at least tepid VUS (PPP ≥ 50%). In contrast, 15/17 (88%) MVs negative for the *CHD8*-IDDAM episignature were initially classified as cool/ice-cold VUS, or with evidence against pathogenicity (PPP < 50%), 1/17(6%) classed as tepid VUS, and 1/17 (6%) as warm VUS (Figs. 1D and 2A, B, Supplementary Table S1, Supplementary Table S4) [36].

Applying ACMG/ACGS criteria PP4/PS2 at strong level for high confidence signatures as per ACGS 2024 guidelines and international reporting recommendations allowed for the reclassification of eight variants from VUS to likely pathogenic, and three variants from likely pathogenic to pathogenic (Supplementary Table S4) [28, 35]. Notably, 7/8 of the missense variants which were upgraded from VUS to likely pathogenic were classified as tepid or warm VUS. Without episignature analysis it is unlikely that sufficient evidence could have been obtained to resolve their clinical significance (Fig. 2C).

Collectively, these data demonstrate a correlation between the ACMG/ACGS predictions and DNAm results outcome, with a posterior probability of pathogenicity of ≥50% strongly correlated with the likelihood of having a CHD8-IDDAM episignature (Fig. 2B, *p* = 0.0002). They also show that episignature analysis can substantially improve classification of *CHD8* variants.

### Pathogenic *CHD8* MVs are in or near functionally important protein regions and domains

**Fig. 3:**
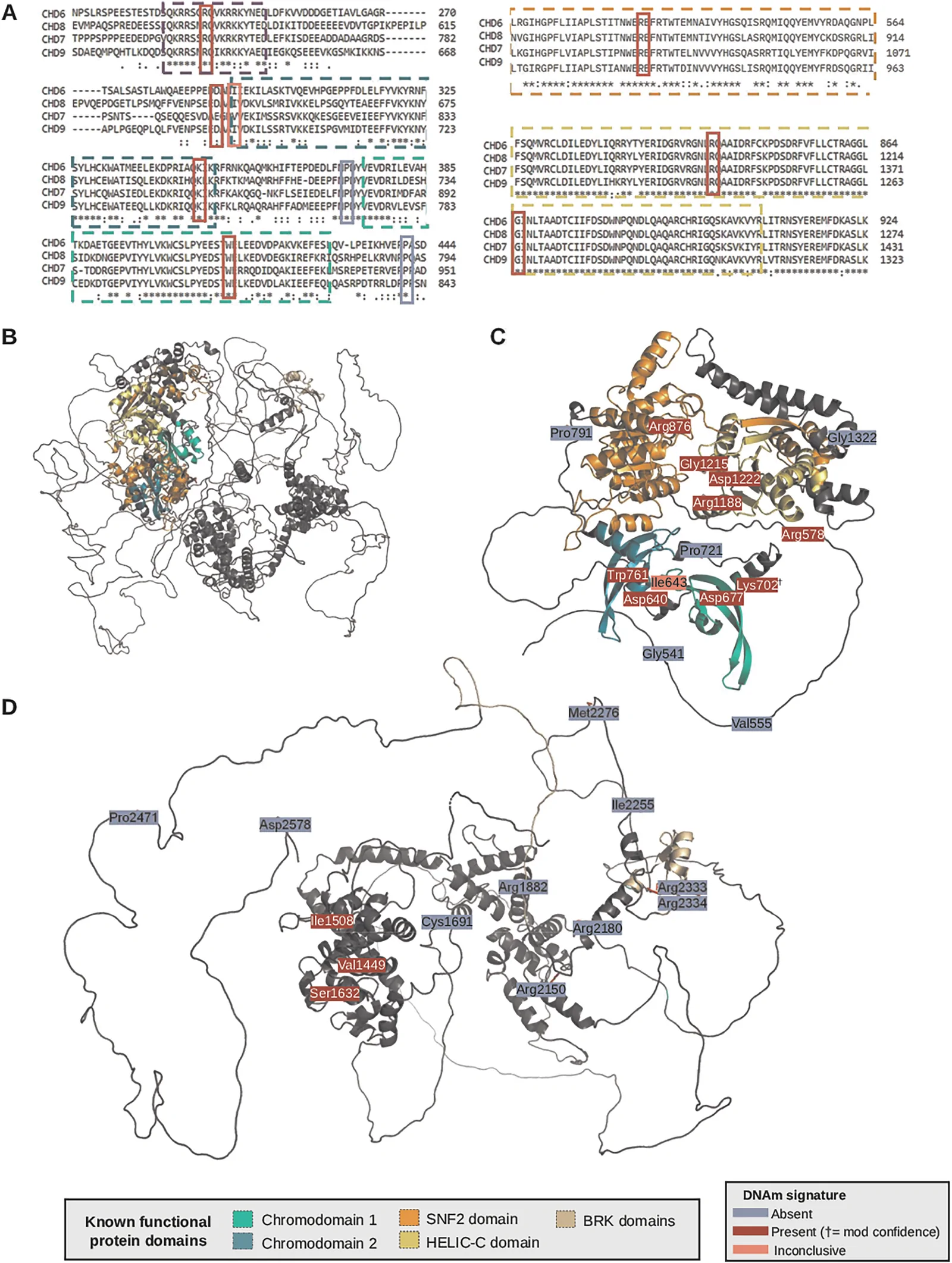
Location and conservation of disease causing *CHD8* missense variants. **A** Alignment of the amino acid sequences of CHD8 paralogs - members of the CHD subfamily III (CHD6,7,8,9). Important functional domains are surrounded by boxes in dashed lines, colours as per the legend at the bottom of the figure. The newly characterised putative N-CRD domain [40] is indicated by a purple dashed box. **B** AlphaFold predicted structure of CHD8 with functional domains coloured. **C**, **D** Location of CHD8 MVs with and without a DNAm signature within AlphaFold predicted protein structure.

The majority of the episignature positive MVs were located within, or adjacent to, known functional protein domains including CHR1, CHR2 and the HELIC-C domain, in highly conserved regions of the protein (Fig. 3A–C, Supplementary Fig. S4). Interestingly, three additional MVs with a high confidence signature, c.4346T>A p.(Val1449Glu), c.4523T>G p.(Ile1508Ser) and c.4895C>T p.(Ser1632Leu) were not located in a known functional domain, but were all in a highly conserved and highly structured region of the protein according to the AlphaFold prediction as well as in close proximity to each other within the structured regions of the protein (Fig. 3D, Supplementary Fig. S4). Finally, one episignature-positive MV, c.1732C>T p.(Arg578Cys), is within a flexible, unstructured (albeit evolutionarily conserved) region of high missense constraint located N-terminal to CRD1 domain (Fig. 3C). Relevantly, N-terminal to Chromodomain (N-CRD) region in *CHD7* has been demonstrated to be essential for the protein’s nucleosome remodelling activity [38]. All the MVs with negative episignatures were projected to be in flexible, unstructured linker domains (Fig. 3, Supplementary Table S1) [36].

### Pathogenic *CHD8* MVs result in phenotype that overlaps with LoF variants, but may be relatively milder

We then compared the clinical features of individuals with pathogenic vs non-pathogenic *CHD8* MVs.

**Fig. 4:**
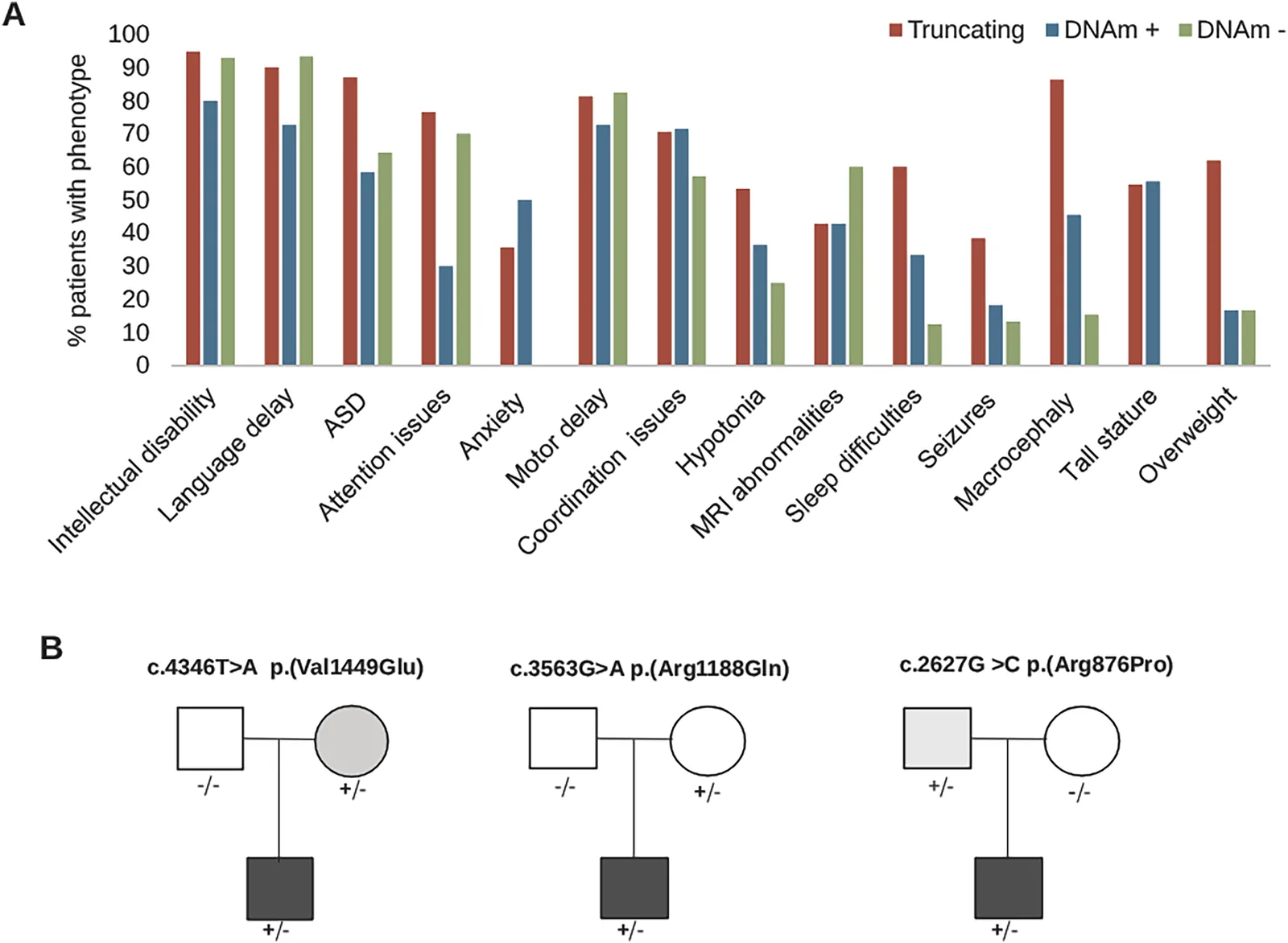
Phenotypes and episignatures of *CHD8* loss of function and missense variants. **A** Phenotypes of patients with DNAm- missense variants, DNAm+ (disease-causing) missense variants and pathogenic LoF variants are variable but clinically similar. **B** Simplified pedigrees of families with inherited *CHD8* MVs, and where parents are mildly or sub-clinically affected. Dark grey stands for affected probands, light grey for mildly affected individuals.

This revealed that a higher proportion of individuals with non-pathogenic *CHD8* MVs had ID, language and motor delay, ASD and attention issues. This is likely explained by reporting bias of the diagnostic laboratories. In contrast, we found that patients with macrocephaly and/or tall stature are enriched in the patients with pathogenic vs non-pathogenic MVs, although this was not statistically significant, likely due to small sample sizes (Fig. 4A, Supplementary Fig. S2).

We next investigated the overall phenotypes of individuals with pathogenic *CHD8* MVs and previously published truncating variants [12, 16] (Fig. 4A). We found two nominally significant differences - a lower frequency of reported attention issues, and of macrocephaly, in individuals with MVs (respectively 30% and 45%) in comparison with individuals with truncating variants (respectively 76% and 86%, *p* = 0.04 and *p* = 0.03), with effect size estimates also supporting this observation, with lower odds of macrocephaly (OR 0.13, 95% CI 0.02–0.72) and attention issues (OR 0.16, 95% CI 0.03–0.81) in individuals with MVs. However, the *p* values did not remain significant after Benjamini–Hochberg correction for multiple testing (both FDR-adjusted *p* = 0.28) (Supplementary Fig. S2). ASD (58% vs 87%), being overweight (17% vs 61%), ID (80% vs 94%), motor delay (73% vs 81%) and hypotonia (36% vs 53%) were also reported less frequently in individuals with MVs, but not anxiety (43% vs 36%). These differences were not statistically significant. Where photographs were available, the facial dysmorphism of individuals with P/LP *CHD8* MVs demonstrated a considerable overlap with those previously reported in individuals with a *CHD8* LoF variant, with shared features including broad forehead with prominent glabella, arched and sparse eyebrows, hypertelorism, down slanting palpebral fissures, full cheeks and prominent chin [12].

In terms of inheritance pattern, of the six probands known to have de novo variants (including both monozygotic twins), only one was episignature-negative, with the remaining five being positive. Where inheritance was unknown, nine patients were negative and four positive. Eight episignature-negative patients had inherited their variant, but more notably three male probands had inherited their *CHD8*-IDDAM episignature-positive MVs c.3563G>A p.(Arg1188Gln), c.4346T>A p.(Val1449Glu), and c.2627G>C p.(Arg876Pro) (the former two maternally, and the latter paternally) (Fig. 1A). The mother of the boy with the c.3563G>A p.(Arg1188Gln) MV was clinically unaffected, and the mother of the boy with c.4346T>A p.(Val1449Glu) MV was originally reported to be unaffected, but was found to be mildly affected on reverse phenotyping [15] (Fig. 4B, Supplementary Table S3). The father of the boy with the c.2627G>C p.(Arg876Pro) MV was reportedly mildly affected.

These results show that individuals with (likely) pathogenic *CHD8* MVs have overlapping clinical features compared to individuals with *CHD8* LoF variants, but overall clinical presentation may be milder in some cases, and that *CHD8* disease-causing MVs can be inherited and can be associated with incomplete penetrance or variable expressivity.

## Discussion

Here, through a systematic analysis of a cohort of individuals with *CHD8* MVs assembled from multiple data sources, we demonstrate that episignatures are useful in resolving the clinical significance of variants in neurodevelopmental conditions with relatively indistinct phenotypes, reduced penetrance, variable expressivity and pleiotropy.

Our analysis also shows that co-occurrence of macrocephaly or tall stature along with neurodevelopmental issues may have a better positive predictive value for *CHD8*-IDDAM in presence of a *CHD8* MV VUS located in or near in or near functionally important protein regions and domains, that is at least ACMG/ACGS tepid (PPP ≥ 50%) (Figs. 2–4). These observations along with the results of the episignature testing also strongly suggest that disease-causing *CHD8* MVs act as loss-of-function or reduced function alleles, rather than as gain-of-function alleles.

Our phenotypic analysis is consistent with previous publications in finding that *CHD8*-associated IDDAM is a disorder with variable and non-specific clinical features [11–14, 16], with comprehensive phenotyping providing limited diagnostic value in the context of resolving variants of uncertain significance. Interestingly, pleiotropy and variable expressivity persisted even within the group of patients with a DNAm signature, now with a presumed genetic diagnosis of *CHD8*-related IDDAM, as well as between unrelated patients with the same MV. We also show evidence for incomplete penetrance. Of note, the c.4346T>A p.(Val1449Glu) variant was initially discounted as a cause of disease due to the variant being inherited from an ‘unaffected’ parent, due to the paucity of published accounts of incomplete penetrance in *CHD8*-related IDDAM. Literature review revealed a similar finding in an affected male proband and unaffected mother carrying a *CHD8* c.4984C>T p.(Arg1662Ter) variant, indicating this may not be restricted to missense variants [39]. Notably, patients with pathogenic variations in *CHD3* and *CHD5* have also been shown to display NDDs with variable expressivity and reduced penetrance and inheritance of variants (both LoF and MVS) from mildly affected/unaffected mothers has been reported [3, 5, 40].

Overall, this work shows the value of episignatures as a tool to resolve the significance of VUS, substantially expands the known genotype spectrum of *CHD8*-related IDDAM and reveals the variability of the associated phenotypes. Future work investigating the correlation between episignature characteristics and disease phenotype, particularly in the context of inherited pathogenic variation and incomplete penetrance in variable neurodevelopmental disorders, could be beneficial to further explore the implications for using episignatures as prognostic and therapeutic markers.

## Supplementary information

Supplementary Figures S1-S4

Supplementary Tables S1-S4

## Data Availability

Data in the EpiSign Knowledge Database, including methylation data from this CHD8 study cohort, are not available due to Research Ethics Board and institutional restrictions. EpiSign™ is proprietary software and is not publicly available. Previously unpublished variants have been submitted to Clinvar, Accession numbers: SCV007596787, SCV007596786, SCV007596784, SCV007596783, SCV007596782, SCV007596781, SCV007596780, SCV007596779, SCV007596778, SCV007596777, SCV007596776, SCV007596775, SCV007596773, SCV007596772, SCV007596771, SCV007596770, SCV007596769, SCV007596768, SCV007596767, SCV007596766, SCV007596765, SCV0075.96764, SCV007596793, SCV007596792, SCV007596791, SCV007596790, SCV007596789, SCV007596788, SCV007596785, SCV007596774, SCV007596763, SCV007596045.
