# Supplementary material for: An episignature informed systematic analysis to ascertain the clinical significance and consequences of CHD8 missense variants
Source: Eur J Hum Genet. 2026 Aug 10;34(11):1654–62. doi: 10.1038/s41431-026-02209-4 (PMC13743776; doi:10.1038/s41431-026-02209-4)
Supplement: Supplementary file 1 [file 41431_2026_2209_MOESM1_ESM.pdf]

Supplementary Figure S1

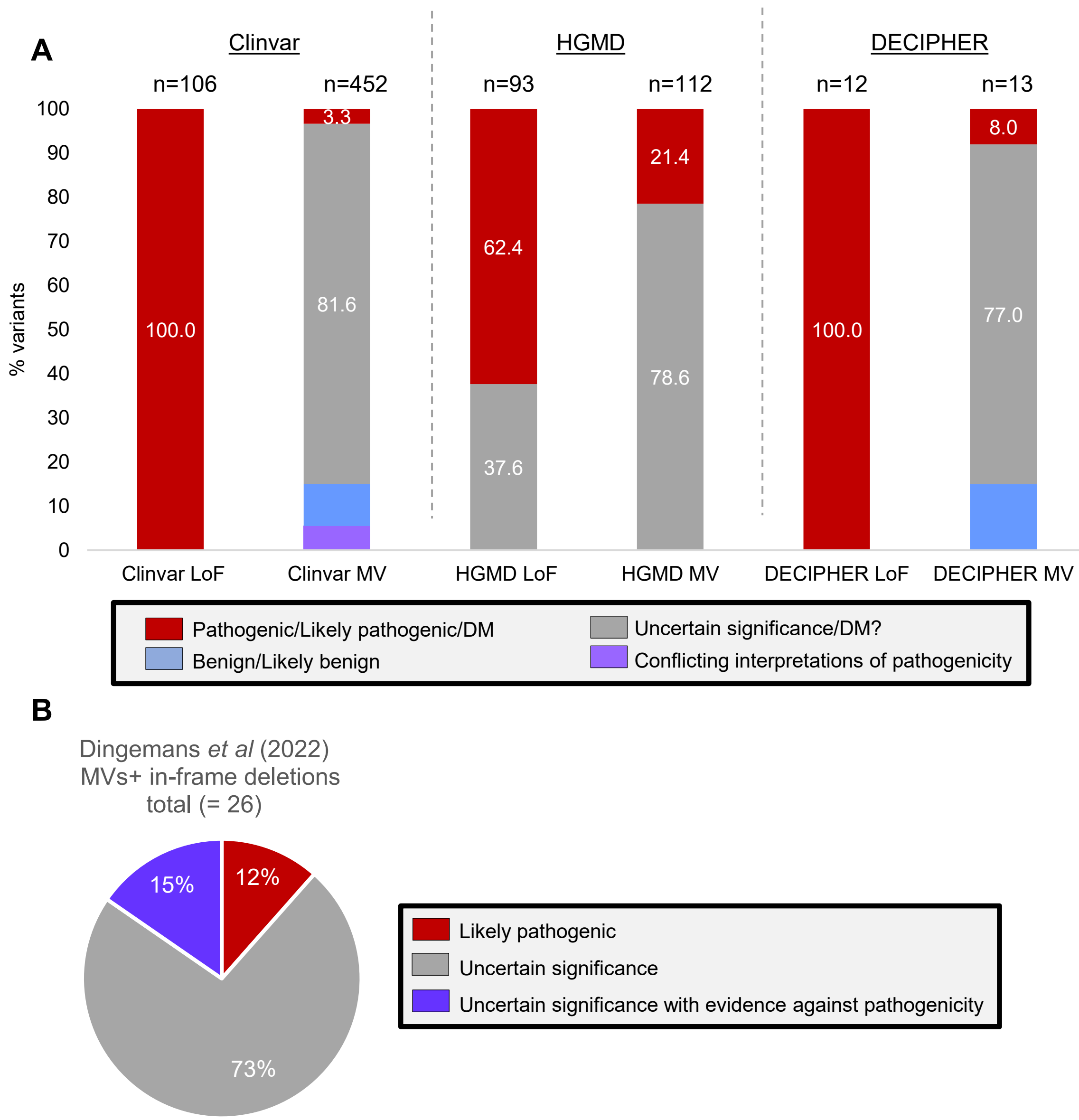

Supplementary Figure 1: Classification of *CHD8* missense variants in a diagnostic setting**.** (A) Distribution of missense and clear loss of function (truncating and nonsense only) variants in *CHD8* by classification as deposited in ClinVar, HGMD and DECIPHER. DM= deleterious mutation (as used in HGMD) (B) Pie chart of missense variants and in-frame deletions in *CHD8* included in[16] classified according to ACMG/ACGS guidelines, details in Supplementary Table S1.

Supplementary Figure S2

A

					DNAm+ vs DNAm –			DNAm+ vs LoF		
Phenotype	Group1 (total =25)	Group2 (total =19)	DNAm+ (total=13)	LoF (total =25)	p value (raw)	p value (corrected)	OR (95% CI)	p value (raw)	p value (corrected)	OR (95% CI)
Intellectual disability	15/19	15/16	8/10	18/19	0.50	0.95	0.69 (0.11–4.20)	0.27	0.63	0.22 (0.02–2.82)
Motor delay	16/21	11/16	8/11	13/16	1.00	1	0.64 (0.12–3.28)	0.66	0.84	0.62 (0.10–3.82)
Language delay	15/19	16/17	8/11	18/20	0.27	0.76	0.87 (0.15–4.91)	0.32	0.64	0.30 (0.04–2.13)
Macrocephaly	6/18	3/16	5/11	19/22	0.18	0.76	1.36 (0.30–6.12)	**0.03**	0.28	**0.13 (0.02–0.72)**
Tall stature	5/13	0/10	5/9	12/22	**0.04**	0.56	**15.9 (0.68–369.4)**	1.00	1	1.04 (0.22–4.96)
Overweight	4/11	2/10	1/6	13/21	1.00	1	0.75 (0.11–5.01)	0.08	0.32	0.12 (0.01–1.25)
ASD	10/20	11/15	7/12	20/23	0.68	0.95	0.80 (0.18–3.46)	0.09	0.32	0.25 (0.05–1.18)
Attention issues	6/12	7/12	3/10	13/17	0.23	0.76	0.60 (0.13–2.80)	**0.04**	0.28	**0.16 (0.03–0.81)**
Anxiety	4/14	1/9	4/8	5/14	0.08	0.56	1.25 (0.27–5.88)	0.66	0.84	1.67 (0.32–8.63)
MRI abnormalities	5/14	7/12	3/7	6/14	0.64	0.95	1.00 (0.17–5.88)	1.00	1	1.00 (0.17–5.88)
Sleep difficulties	5/13	2/12	3/9	12/20	0.58	0.95	0.83 (0.17–3.94)	0.25	0.63	0.33 (0.06–1.73)
Coordination issues	6/13	5/9	5/7	12/17	1.00	1	1.20 (0.23–6.29)	1.00	1	1.04 (0.15–7.27)
Hypotonia	6/22	3/16	4/11	8/15	0.68	0.95	0.91 (0.19–4.33)	0.45	0.7	0.50 (0.10–2.46)
Seizures	3/21	2/17	2/11	5/13	1.00	1	0.71 (0.12–4.24)	0.39	0.68	0.36 (0.05–2.37)

B

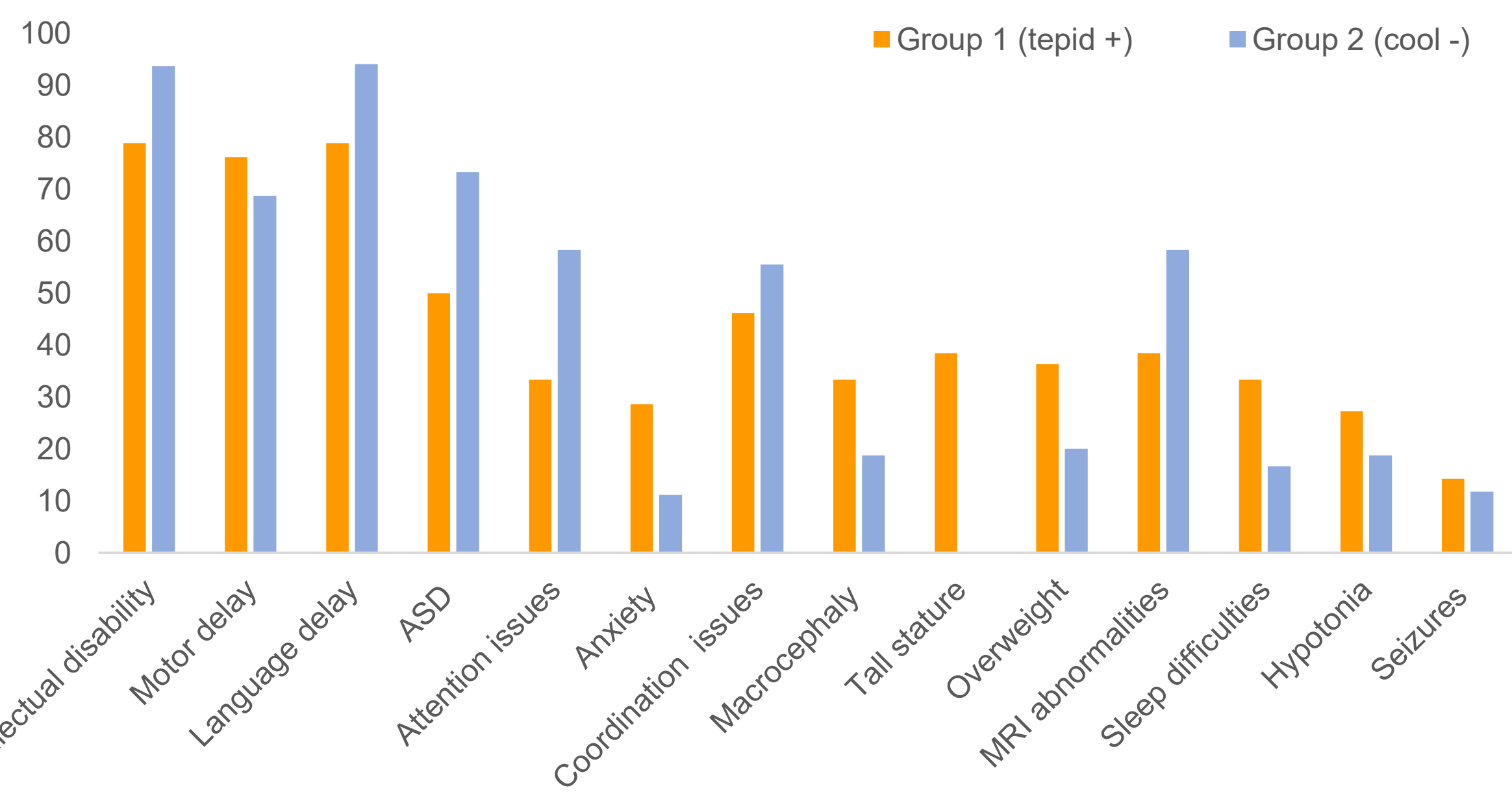

Supplementary Figure S2: Clinical features of patients used for phenotypic analyses (A) Number of patients for whom the indicated phenotypic information was provided, divided into patients in group 1 (at least tepid VUS) and 2 (cool VUS/evidence against pathogenicity), patients with a positive and negative CHD8 DNAm signature (including patients who had an identical variant to another patient where a positive signature was identified but where DNA was unavailable for analysis), and LoF patients, and results of statistical analyses. (B) Phenotypic comparison between patients in group 1 and group 2

Supplementary Figure S3

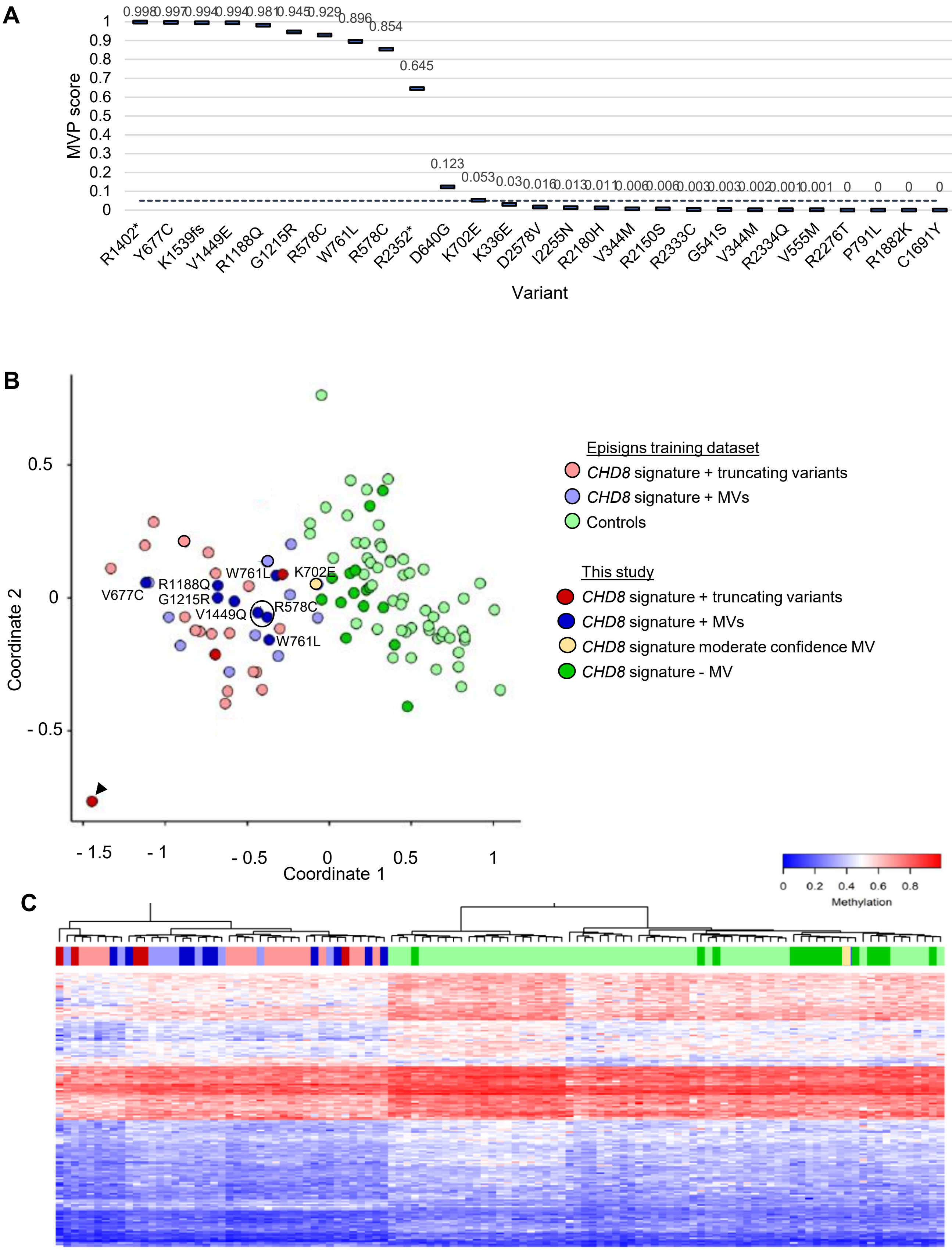

Supplementary Figure S3: DNAm analysis according to the EpiSsigns pipeline for patients with *CHD8* MV's and LoF variants shows that MVs behave similarly to pLoF variants according to all metrics used for Episignature analysis, including MVP scores (A), MDS analysis (B) and heatmap hierarchical clustering (C). Note that for MVP scores results closer to 1 indicate samples that are more similar to the *CHD8* episignature samples, results closer to 0 indicate samples that are more similar to control samples, with the cutoff for significance according to the EpiSigns pipeline being >0.05 (dotted line in A). The identity of the MVs for the patients in our cohort is indicated on the MDS plot. The arrowhead indicates a pLoF variant where atypical clustering of was observed on the heatmap and MDS plot likely due to the very young age of the patient as the sample was collected at 4 days old. The heatmap is colour coded as per the legend above in B.

Supplementary Figure S4

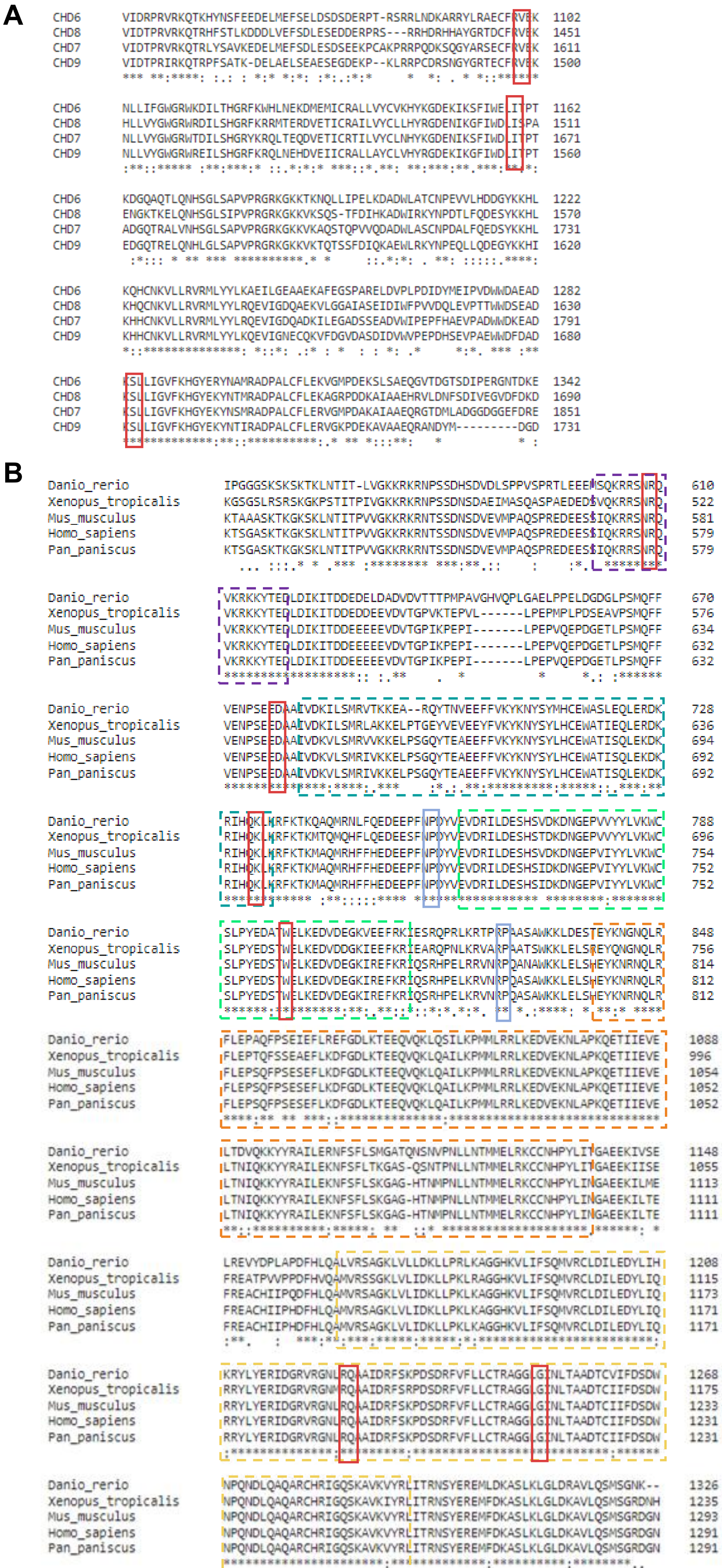

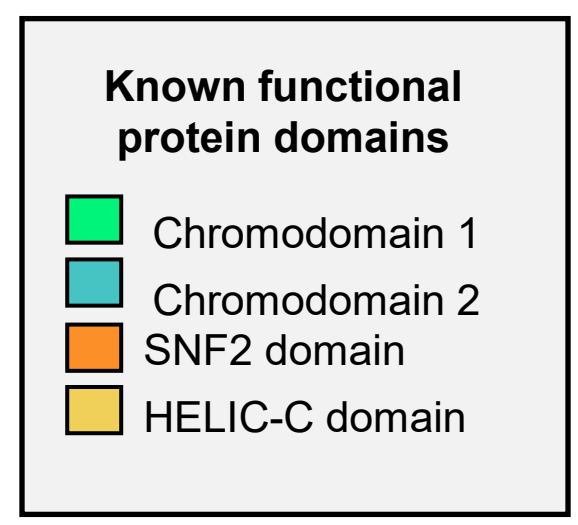

Supplementary Figure S4: Pathogenic missense variants are located in highly conserved functionally important protein domains. (A) Alignment of the amino acid sequences of CHD8 paralogs - members of the CHD subfamily III (CHD6,7,8,9). (B) Alignment of the amino acid sequence of CHD8 homologs in distantly related vertebrates indicates that pathogenic CHD8 missense variants are highly conserved, and located in highly conserved regions. Important functional domains are surrounded by boxes in dashed lines, colours as per the legend at the side of the figure. The N-CHD domain is indicated by a purple dashed box. Pathogenic CHD MV's are in red boxes and non-pathogenic MV's in blue boxes.
